# MicroRNA Signatures as Future Biomarkers for Diagnosis of Diabetes States

**DOI:** 10.3390/cells8121533

**Published:** 2019-11-28

**Authors:** Srividya Vasu, Kenjiro Kumano, Carly M. Darden, Irum Rahman, Michael C. Lawrence, Bashoo Naziruddin

**Affiliations:** 1 Islet Cell Laboratory, Baylor Scott and White Research Institute, 3434 Live Oak Street, Dallas, TX 75204, USA; srividya.vasu@bswhealth.org (S.V.); kenjiro.kumano@bswhealth.org (K.K.); Carly.Darden@bswhealth.org (C.M.D.); irumrahman97@gmail.com (I.R.); michael.lawrence@bswhealth.org (M.C.L.); 2 Department of Biomedical Studies, Baylor University, 1301 S. University Parks Dr., Waco, TX 75706, USA; 3 Annette C. and Harold C. Simmons Transplant Institute, Baylor University Medical Center, 3410 Worth St., Suite 950, Dallas, TX 75246, USA

**Keywords:** diabetes, biomarker, miRNA, islet

## Abstract

Diabetes results from the inability of pancreatic islets to maintain blood glucose concentrations within a normal physiological range. Clinical features are usually not observed until islets begin to fail and irreversible damage has occurred. Diabetes is generally diagnosed based on elevated glucose, which does not distinguish between type 1 and 2 diabetes. Thus, new diagnostic approaches are needed to detect different modes of diabetes before manifestation of disease. During prediabetes (pre-DM), islets undergo stress and release micro (mi) RNAs. Here, we review studies that have measured and tracked miRNAs in the blood for those with recent-onset or longstanding type 1 diabetes, obesity, pre-diabetes, type 2 diabetes, and gestational diabetes. We summarize the findings on miRNA signatures with the potential to stage progression of different modes of diabetes. Advances in identifying selective biomarker signatures may aid in early detection and classification of diabetic conditions and treatments to prevent and reverse diabetes.

## 1. Introduction

Pancreatic islets regulate glucose homeostasis through insulin and glucagon, which are central to key biological processes and energy homeostasis. Loss or impairment of islet function results in dysregulated blood glucose, which gives rise to multiple life-threatening complications including cardiovascular disease, neuropathy, nephropathy, blindness, and stroke. High blood glucose, the most common clinical sign of diabetes, is usually observed only when islet beta cells are already deficient or exhausted. In the case of type 1 diabetes (T1D), clinical symptoms manifest when >80% of islet beta cells are already lost to autoimmunity. During the progression of type 2 diabetes (T2D), islet compensation maintains normoglycemia for years without symptoms before progressing to a state of detectable impaired glucose tolerance (IGT) and impaired fasting glucose (IFG). In addition, during pregnancy, lack of islet compensation leads to gestational diabetes (GDM), which increases risk for T2D. Between 24% and 62% of people with diabetes are unaware of the disease, undiagnosed, and untreated [1], suggesting a large gap in current diagnostic practices. Early detection of islet cell stress preceding loss of islet function would allow for therapeutic interventions to delay or alleviate the onset of diabetes.

Emerging technologies to recover, amplify, and detect nucleic acids in the blood have allowed for sensitive methods to make correlations of gene expression profiling with specific states of disease. Several studies suggest that there is selective expression of circulating microRNAs (miRNA) that may correlate with diabetic conditions. In this review, we have surveyed and evaluated a compilation of studies regarding the use of miRNAs as potential biomarkers to improve current diagnostic practices performed in the clinic to detect and monitor diabetes. Due to complexity and variability of blood-borne miRNAs arising from multiple tissue sources during metabolic and inflammatory disease, we conclude that singular miRNAs may not be sufficient to precisely or accurately diagnose pre-diabetic (pre-DM) states or conditions. However, we propose that correlations of groupings of miRNAs that are selective for components related to inflammatory, immune, and metabolic stress would provide insight to progression of various modes of diabetes. These microRNAs could arise from brain, liver, muscle, adipose, myeloid, lymphoid, and islet cells among other tissue types contributing or responding to pre-DM conditions. Future studies focused on identifying and validating such groupings or “signatures” of miRNAs may prove useful in revealing the landscaping of disease progression for diagnosing or treating patients in the pre-DM state.

### 1.1. Current Diagnostic Practices

During routine annual health check-ups across various healthcare systems globally, fasting and postprandial glucose levels, glycated hemoglobin A1c (HbA1c), and symptoms reported by patients are assessed as first-step diagnostic measures of diabetes. Additionally, several questionnaires including Finnish Cardiovascular Risk Study (FINRISK), Australian type 2 diabetes risk assessment tool (AUSDRISK), and The Indian Diabetes Risk score (IDRS) have been developed to screen for risk of undiagnosed diabetes [2,3,4]. GDM is usually diagnosed during a routine glucose tolerance test between 24 and 28 weeks of pregnancy. Metabolic outcomes after islet transplantation are routinely monitored using fasting and stimulated C-peptide, glucose, and HbA1c levels. Even though these diagnostic findings help establish overt diabetes or assess islet graft function after transplantation, they do not indicate beta-cell stress or death during or before the pathological events. In addition, C-peptide, although used for assessing beta-cell function, is also released upon islet damage and thus cannot distinguish islet damage from intact function. Diagnosing beta-cell stress before irreversible loss of beta-cell mass is of prime importance for efficient therapeutic interventions.

### 1.2. Search for New Biomarkers

In T1D, autoantibodies to islet antigens–insulin (IAA), glutamic acid decarboxylase 65 (GAD), islet cell cytoplasmic antigens (ICA), zinc transporter 8 (ZnT8), and protein tyrosine phosphatase-like protein (IA-2/ICA512) [5] are validated biomarkers of autoimmunity. These biomarkers, along with C-peptide levels, can provide an indication of autoimmunity and beta-cell function, respectively, but cannot be used to measure the onset and rate of beta-cell death. In addition, these tests are performed in the clinic to distinguish T1D from T2D only after patients report diabetes symptoms and not for diagnostic purposes or for non-diabetic individuals [6].

Currently, no non-invasive biomarker is used clinically for early detection of islet stress and dysfunction before loss of beta-cell mass and the resulting changes in clinical parameters, including decreased C-peptide levels, increased HbA1c, and fasting and 2 h postprandial hyperglycemia. Testing biomarkers of islet cell stress and damage would be a simple and noninvasive technique for annual health check visits. Ideally, diagnostic biomarkers for diabetes should meet the following criteria to be: highly selective and specific, neutral to normal metabolic and physiological changes, easily detectable in circulation, highly reproducible, and non-invasive. Most importantly, a biomarker should be detectable in circulation before onset of irreversible loss of beta-cell mass.

In the search for biomarkers of islet stress, damage, and death, circulating cell-free DNA (cfDNA), advanced glycation end products, isoprostanes, advanced oxidation protein products, oxidative DNA damage markers, branched chain amino acids, and short-chain fatty acids have been investigated [7,8,9]. However, each of these measures have their challenges in meeting the aforementioned biomarker criteria. For example, insulin cfDNA reflects beta-cell death and is elevated in response to autoimmunity in NOD mice [10], recent-onset T1D [11,12], and islet transplant 24 h after islet infusion [13]. However, insulin cfDNA is not useful in predicting beta-cell death prior to the pathological events. Advanced glycation end-products, including HbA1c, on the other hand, reflect the effects of chronic hyperglycemia, which are useful in monitoring glucose control in diabetic patients. Other markers including oxidative DNA damage markers and advanced oxidation protein products are a result of chronic oxidative stress and implicated in diabetes pathogenesis. However, chronic oxidative stress is also implicated in other diseases, including cancer, Parkinson’s disease, and Alzheimer’s disease, and thus is non-specific. Branched chain amino acids and short-chain fatty acids are influenced by exercise and dietary changes (acute starvation or low-protein diets), maple syrup urine disease, and possibly hypermetabolic states such as sepsis, injury, or cancer [14].

### 1.3. miRNAs as Biomarkers

miRNAs are specialized short non-coding RNAs (20–22 nt) that inhibit target mRNA translation. Recent research on circulating miRNAs highlights their usefulness as biomarkers of diseases. Under various conditions, cells release miRNAs that are free or in microvesicles that can be taken up by other cell types. These extracellular miRNAs are important mediators of cell-to-cell communication and coordinate biological functions including angiogenesis, tumor cell invasion, and immune response. miRNAs are reliable biomarkers due to their resistance to degradation in circulation and their enrichment in particular tissues, which reveals the source of circulating miRNAs [15]. Modern technologies in nucleic acid amplification, sequencing, and analysis have allowed us to identify miRNAs produced and released by islets under stress conditions. For this review, we have compiled existing RNA sequencing, microarrays and quantitative polymerase chain reaction (qPCR) data from comprehensive studies in humans and present the potential use of miRNA signatures for future use in detecting pre-DM and/or staging progression and mode of diabetes.

## 2. Methods

To locate relevant articles, we searched PubMed, beginning with the following keywords: ((mir) or (miRNA) or (circulating mir) or (circulating miRNA)) and ((biomarker) or (biomarkers)) and ((diabetes) or (islet) or (beta)). The second search included the following search keywords: ((miRNA [Title/Abstract]) or (microRNA[Title/Abstract])) and ((diabetes[Title/Abstract]) or (obesity) or (prediabetes)) and (biomarker *). We filtered the results for clinical studies, trials, comparative studies, evaluation studies, journal articles, meta-analyses, multicenter studies, reviews, systematic reviews, and validation studies in the date range of 2009 to 2019, including only studies with human subjects. In addition, we excluded studies that used whole-blood miRNA analyses instead of plasma or serum analyses.

## 3. Results and Discussion

### 3.1. miRNAs as Biomarkers in Type 1 Diabetes (T1D)

**Figure 1:**
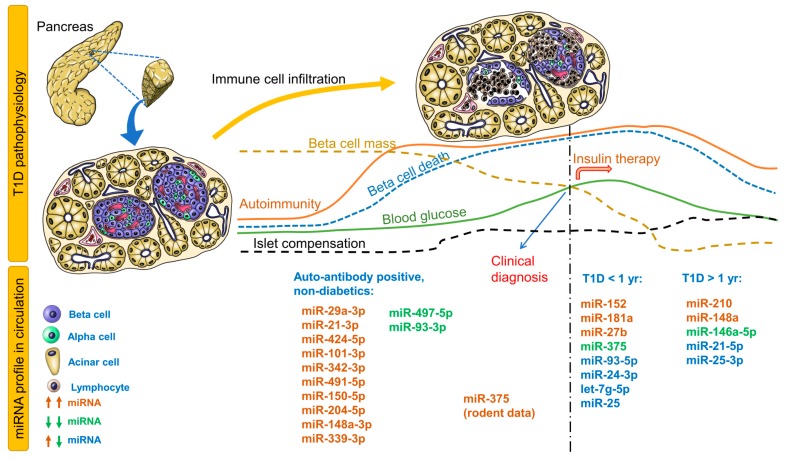
Circulating miRNA profiles at different stages of type 1 diabetes (T1D) pathophysiology. Changes in autoimmunity, beta-cell mass/death, blood glucose, and islet compensation over the course of T1D pathophysiology are provided as line profiles. Circulating miRNA profiles consistent in at least two studies are provided for T1D (<1 and >1 year). All miRNAs identified in autoantibody-positive non-diabetic children before onset of T1D are provided. Upregulated miRNAs are shown as brown; downregulated miRNAs, green; and upregulated or downregulated, blue.

Table 1 provides circulating miRNA profiles for patients with recent-onset T1D, T1D > 1 year, maturity-onset diabetes of the young, latent autoimmune diabetes in adults, and individuals at risk for T1D (autoantibody positive). Immune cell infiltration is the first event that leads to significant reduction in beta-cell mass and eventual hyperglycemia, followed by changes in blood glucose, autoimmunity, beta-cell mass, beta-cell death, islet compensation, and corresponding circulating miRNA profiles (Figure 1). For the autoantibody-positive non-diabetic group, all miRNAs identified in 4 studies are provided in Figure 1. For recent-onset and longstanding T1D groups, only those miRNAs that were identified independently in at least 2 studies are provided in the figure. Any correlations of miRNAs to metabolic parameters are provided in Supplementary Table S1. It is evident that distinct groups of miRNAs can be identified for non-diabetic individuals vs. those with T1D.

In non-diabetic children (autoantibody positive) who participated in the T1D TrialNet Pathway to Prevention study, children with multiple autoantibodies and elevated miR-29a-3p, miR-21-3p, and miR-424-5p were more likely to progress to T1D within 2 years of follow-up [16]. In another cohort of non-diabetic children with islet autoantibodies, circulating miR-339-3p was elevated while miR-497-5p was decreased [17]. In the high-risk group, miR-148a-3p was significantly elevated while miR-93-3p was decreased in high-HLA-risk children. Interestingly, miR-342-3p correlated negatively while miR-144-5p correlated positively to insulinoma-2 antigen (IA2A) antibody titers. miR-378a-3p correlated negatively to IA2A and ZnT8A(Trp) but positively with GAD autoantibodies [17]. In a different autoantibody-positive cohort, miR-101-3p was elevated only in non-diabetic children with multiple autoantibodies and in children with recent-onset T1D and also correlated positively with GAD autoantibody levels [18]. In this cohort, miR-204-5p was elevated only in children with recent-onset T1D but not in non-diabetic autoantibody-positive children. In contrast, miR-204-5p was elevated in autoantibody-positive, non-diabetic children and recent-onset T1D. miR-204-5p levels also demonstrated good ability to distinguish autoantibody-positive non-diabetic children from recent-onset T1D children [19]. Of these miRNAs, miR-29a-3p, miR-342-3p, miR-148a-3p, and miR-93-3p were identified in studies involving recent-onset T1D children, whereas miR-424-5p, miR-101-3p, miR-148a-3p, and miR-93-3p were identified in studies involving children with longstanding T1D (>1 year). These studies in non-diabetic autoantibody-positive children should be validated independently across multiple institutions to evaluate the potential of these miRNAs as early predictive biomarkers of T1D.

In patients with recent-onset T1D, the most consistently upregulated miRNAs were miR-152, miR-181a, and miR-27b (Figure 1, Table 1), while miR-375 was consistently down regulated in independent cohorts (Figure 1, Table 1). miR-25, miR-24-3p, let-7g-5p, and miR-93-5p were either upregulated or downregulated in various recent-onset T1D cohorts. Apart from these miRNAs, a number of miRNAs displayed a positive or negative association with glycemic parameters (Table S1), some of which are described here. In a Danish Remission Phase cohort (European), miR-25 levels 1 month after diagnosis correlated negatively with HbA1c but positively with C-peptide levels at 3 months [20], suggesting an association with residual beta-cell function. However, any association was lost between miR-25 levels measured 12 months after disease onset and glycemic control parameters, possibly due to lack of residual beta-cell function and insulin therapy. In the same cohort after 5-year follow-up, miR-24-3p, miR-146a-5p, miR-194-5p, miR-197-3p, miR-301a-3p, and miR-375 but not miR-25 (although differentially expressed), measured 3 months after diagnosis, predicted stimulated C-peptide, HbA1c, or insulin dose-adjusted HbA1c (IDAA1c) 6 or 12 months after diagnosis [21]. Either upregulated or downregulated miR-25 levels have been reported in recent-onset T1D and longstanding T1D [17,22,23], possibly reflecting differences in residual beta-cell function in these cohorts.

miR-375, an established marker of beta-cell death, was downregulated in a cohort of recent-onset T1D patients [24], did not differ significantly compared to healthy controls in another cohort [25], but was upregulated in another cohort with at least 5 years of disease [26] (Table 1). In the Danish Remission Phase cohort that completed the 5-year follow-up, miR-375 correlated negatively with C-peptide levels 6 months after diagnosis [20]. T1D patients were best classified using miR-375 and miR-21 levels [26].

These conflicting observations are not surprising for at least three reasons: (1) the bulk of beta-cell death in T1D precedes clinical diagnosis, and hence any increase in circulating miR-375 is possibly missed at the time of sampling; (2) the degree, extent, and dynamics of beta-cell death differ between individuals; and (3) islet compensatory mechanisms and ongoing autoimmunity may influence circulating levels even after disease diagnosis. For instance, our observations in mice suggest that miR-375 levels are significantly elevated in circulation immediately (3–6 h) after streptozotocin administration, subsiding to undetectable levels within 24 h. In the context of islet transplantation, we observed that miR-375 levels increase markedly during islet autotransplantation in patients receiving total pancreatectomy with islet auto transplantation, but normalized to baseline levels 7 days after transplantation [35]. In mice, we also observed elevated miR-375 levels 24 h after human islet transplantation, indicating islet inflammation and damage in the peritransplant period [35]. Thus, miR-375 is a useful marker for islet cell damage during transplantation. In the context of diabetes prediction, miR-375 can be used alongside other biomarkers and not as a standalone biomarker.

In recent-onset T1D (<5 years), miR-200a-3p and miR-155-5p correlated negatively with HbA1c and IDAA1c levels [31]. This association was lost in patients with more than 5 years of T1D. Alterations in circulating miRNA profiles were also reported in another cohort, where miRNAs with elevated levels at baseline or 1 year after diagnosis were undetectable 4 to 8 years after diagnosis [27]. In fact, as depicted in Figure 1 and mentioned earlier, miRNAs in autoantibody-positive non-diabetic children were identified in independent studies involving recent-onset T1D or children with longstanding T1D (but not consistently in at least 2 studies, thus missing our threshold for inclusion in Figure 1). Nevertheless, these profiles of circulating miRNAs at different stages of T1D pathophysiology (Figure 1), especially those expressed during early stages of autoimmunity, are attractive candidates that should be validated further in international cohorts for diagnostic utility.

### 3.2. miRNAs as Biomarkers for Pre-Diabetes (Pre-DM) and T2D

**Figure 2:**
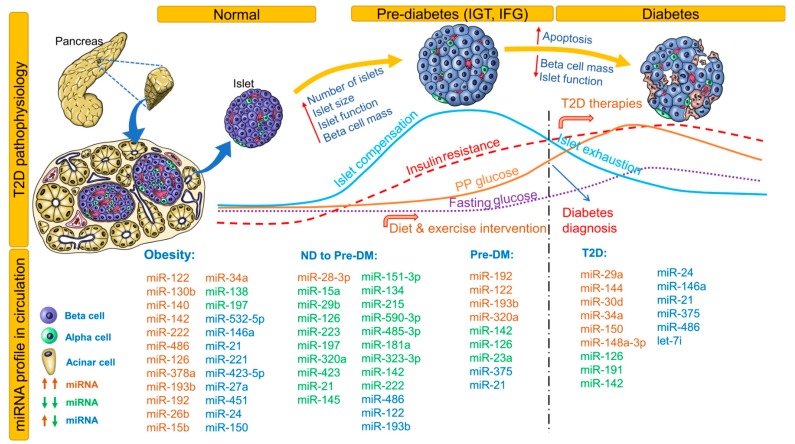
Circulating miRNA profile at different stages of type 2 diabetes (T2D) pathophysiology. Changes in autoimmunity, beta-cell mass, blood glucose, and islet compensation over the course of T2D pathophysiology are provided as line profiles. Circulating miRNA profiles identified and consistent in at least 2 clinical studies are provided. All identified miRNAs are provided for non-diabetic (ND) to pre-DM stage. Upregulated miRNAs are shown in red; downregulated miRNAs, green; and upregulated or downregulated in at least 2 studies, blue. DM indicates diabetes mellitus; IFG, impaired fasting glucose; IGT, impaired glucose tolerance; ND, non-diabetic; PP, postprandial; T2D, type 2 diabetes.

Changes in fasting and postprandial glucose, insulin resistance, islet compensation, and circulating miRNA profiles in the pathophysiology of T2D are shown in Figure 2. This figure also lists all miRNAs identified in non-diabetic healthy individuals who proceeded to develop pre-DM or T2D. Notably, miRNAs identified independently in at least 2 studies for obesity, pre-DM, and T2D are included. For an extensive list of all differentially expressed miRNAs, see Table 2 and Table 3. Association (positive or negative) of differentially expressed miRNAs with metabolic parameters are provided in Supplementary Table S1. Here, we provide a brief review of these correlation analyses conducted in different ethnic populations.

Case-control studies conducted in healthy populations with follow up for future development of pre-DM or T2D provide insights on circulating miRNA profiles before apparent changes in glucose homeostasis and onset of irreversible loss of beta-cell mass. For example, in healthy children aged 7 years, levels of miR-221, miR-28-3p, miR-142-3p, miR-486-3p, and miR-486-5p can be used for risk estimation and obesity classification [36]. In non-diabetic healthy adults, baseline miR-122, miR-15a, miR-197, miR-320a, miR-423, and miR-486 levels were inversely associated with progression to glycemic impairment at 2.5-year follow-up [37]. In another cohort of non-diabetic healthy adults, miR-320a and miR-486-5p increased the odds while miR-375 decreased the odds of insulin resistance [38]. In the Bruneck study cohort, normoglycemic individuals who developed T2D over 10 years were appropriately classified as diabetics based on baseline levels of miR-15a, miR-126, miR-320, miR-223, and miR-28-3p [39]. Thus, the assessment of miRNA signatures instead of a singular miRNA profile can help in not only accurately classifying DM but also distinguishing DM from other diseases.

In a Han Chinese non-diabetic cohort, low miR-126 levels predicted future development of T2D [40]. In another healthy cohort, miR-126, miR-148a, and miR-375 correlated negatively to glucose area under the concentration curve values, miR-29a and miR-21 correlated positively to homeostatic model assessment of insulin resistance (HOMA-IR) values, and miR-29a correlated positively to homeostasis model assessment of β-cell function (HOMA-B) values [26]. In a European relationship between insulin sensitivity and cardiovascular disease risk (RISC) study cohort of non-diabetic, normotensive individuals who proceeded to develop pre-DM at 3-year follow-up, miR-181a, miR-323-3p, miR-342-3p, miR-222, miR-483-5p, miR-151-5p, miR-532-3p, miR-142-5p, miR-625, miR-27b, and miR-590-3p were prognostic and diagnostic biomarkers of beta-cell dysfunction. In this group, a miRNA signature of reduced miR-21, miR-145, miR-151-3p, miR-134, miR-215, miR-590-3p, miR-485-3p, miR-181a, and miR-323-3p was diagnostic of beta-cell dysfunction [41]. In a general population study of 1000 individuals, elevated miR-122 levels correlated with liver enzymes, adiposity, inflammation, insulin resistance, and an adverse lipid profile. Treatment with atorvastatin reduced serum miR-122 levels significantly. Over a period of 15 years, individuals with a higher miR-122 level at baseline were at increased risk of developing metabolic syndrome or T2D [42]. Overall, from these studies, levels of miR-28-3p, miR-142, miR-486, miR-122, miR-15a, miR-320a, miR-126, and miR-375 appear to be consistently (in different cohorts) altered in circulation even before the onset of any clinical symptoms. Further studies are warranted in healthy populations to validate these findings and to discover consistent and unique circulating miRNA signatures to predict future development of T2D.

Obesity increases the risk for development of T2D/metabolic syndrome and is termed ‘diabesity’ (obesity-induced diabetes). Although not the focus of our review, we provide a brief overview of miRNAs associated with obesity traits (Figure 2) and related effects on glycemic status. In Caucasian obese children, increased miR-486-5p/miR-486-3p, miR-142-3p and HOMA-IR, together with decreased miR-28-3p, explained about 60% of variance in body mass index [36].

In obese Europeans, miR-144-5p, let-7d, miR-34a, and miR-532-5p strongly predicted insulin resistance [62]. For other associations, see Supplementary Table S1. Interestingly, in patients with metabolic syndrome enrolled in the Practicing Restorative Yoga vs. Stretching for the Metabolic Syndrome (PRYSMS) study, weight loss improved the circulating miRNA profile [59]. In another cohort with obesity, an acute aerobic intervention in a single session further increased circulating levels of miRNAs involved in inflammation [66]. In contrast, in a different cohort participating in the Centro Universitario Ricerca Interdipartimentale Attività Motoria (CURIAMO) trial, a 3-month exercise intervention markedly reduced the levels of miR-146a-5p, also correlating with decreases in total cholesterol and waist circumference [72]. In patients who underwent bariatric surgery, miR-125b, miR-378a, miR-192, miR-629, miR-22-5p, and miR-15a levels were reduced [65]. Similar reductions in circulating miRNAs (although different miRNAs) were observed in an independent cohort after bariatric surgery [74]. Thus, miRNA profiles are helpful in understanding and tracking the efficacy of intervention programs.

As depicted in Figure 2, progressive insulin resistance induces islet compensation that maintains glucose homeostasis. Progression of the disease results in an abnormal glycemic state, characterized by a reduction in beta-cell secretory capacity. This abnormal glycemic state is represented either by IFG or IGT. However, individuals may exhibit both states, thus reflecting a heterogeneous pathogenesis of T2D. Despite impairment in glycemic states, it takes years for patients to progress to overt T2D, and the disease is often undiagnosed due to infrequent/insufficient analysis of both of these parameters. Moreover, distinguishing pre-DM patients from healthy populations has proven difficult due to fluctuations in the tested ranges in clinical parameters and the influence of lifestyle factors. In this regard, even though the timeline of progression from pre-DM to T2D varies, distinct miRNA signatures can be used to predict future development of T2D. In the Bruneck study cohort, miR-126 remained a significant predictor of T2D, with a gradual decrease in levels from controls to those with IFG/IGT to those with T2D. T2D cases can be correctly classified using a panel of miRNAs including miR-15a, miR-126, miR-320, miR-223, and miR-28-3p [39]. In two other cohorts, miR-126 had an inverse association with fasting glucose, HbA1c, and 1 h postprandial glucose [46,56]. In newly diagnosed T2D and pre-DM patients, low levels of serum miR-126 increase the odds ratio for T2D and can distinguish T2D from controls [47]. Pre-DM patients were best distinguished from healthy populations by using a binary random forest classifier based on levels of miR-146a, miR-126, miR-30d, and miR-148a [26]. In T2D patients, miR-486, miR-146b, and miR-15b correlated positively with fasting plasma glucose, and these biomarkers could be used to discriminate T2D patients from healthy controls [67]. Pre-DM patients with higher levels of miR-150 and miR-30a-5p or lower levels of miR-375 and miR-15a were at a higher risk of developing T2D [69]. A miRNA panel consisting of miR-7641-3p, miR-136-5p, miR-490-3p, miR-501-5p, miR-127-5p, miR-4532-5p, miR-483-5p, and miR-210-3p distinguished obese nonprogressors from progressors who developed T2D at 5-year follow-up [77]. Thus, using a panel of miRNAs and other clinical parameters including HbA1c to classify the health status of patients may help in the discovery of the disease before onset.

Use of biomarkers may help not only in predicting future development of disease, but also in monitoring intervention efficiency. For example, in pre-DM patients enrolled in the CORDIOPREV study, elevated miR-150 and miR-130a-5p levels were associated with a decrease in the disposition index (insulin signaling and release); elevated miR-150 levels were associated with a decrease in the insulin sensitivity index and muscle insulin sensitivity index; and elevated miR-375 levels were associated with a decrease in the hepatic insulin resistance index after 4-year follow-up [69]. In another group of pre-DM patients, miR-192 and miR-193b correlated positively with serum triglycerides and fatty liver index. An exercise intervention program significantly reduced these levels over 6 weeks [51]. In patients with metabolic syndrome, after a 3-month weight loss intervention, circulating levels of miR-326, miR-24, miR-425, and miR-652 increased while circulating levels of miR-106b, miR-140, miR-20b, miR-363, miR-486, miR-532, miR-92a, miR-93, and miR-let7c decreased. Among these, changes in miR-146a, miR-151a, miR-23a, miR-181b, miR-181d, miR-21, miR-221, miR-222, miR-223, miR-23, miR-24, and miR-27b levels strongly correlated with weight change over 3 months. Baseline levels of miR-143, miR-145, miR-146, miR-191, miR-221, miR-23a, miR-29a, and miR-584 strongly correlated with weight change after 3 months [59]. A vitamin D–induced increase in miR-152 levels correlated negatively with HbA1c, while a decrease in miR-192 levels correlated positively with fasting glucose in individuals at risk for T2D [63]. In a Chinese cohort with IFG, upregulated levels of miR-144, miR-20a, and let-7b increased the risk for T2D and positively correlated with HOMA-IR, while decreased levels of miR-142 increased the risk for IFG and negatively correlated with HOMA-IR [70].

Irrespective of disease status (pre-DM, T2D, non-diabetic controls), in a cohort of 871 subjects, miR-144-5p correlated inversely with insulin levels, updated homeostasis model assessment (HOMA2) values, and triglyceride levels. miR-122-5p, miR-184, and miR-339-3p were associated with insulin and HOMA2. miR-144-5p, miR-146b-5p, miR-221-3p, miR-642a-5p, and miR-181a-2-3p correlated positively and miR-148a-3p, miR-15-3p, miR-93-5p, and miR-18-3p correlated inversely with HbA1c. In individuals with IFG, in addition to the trend in the whole population, miR-122-5p was independently associated with insulin and HOMA2 index while miR-146b-5p was associated with HbA1c. In addition, miR-885-5p and miR-106b-5p correlated positively with serum glucose levels [95]. These general population studies are important for understanding the associations of miRNAs to metabolic parameters regardless of disease status.

The ability of miRNA signatures to predict pre-DM or T2D should be tested in various ethnic populations. It is possible that specific miRNA signatures may be driven by ethnicity-related factors (genetic or environmental). For example, in a Swedish cohort, miR-15a, miR-29b, miR-24, miR-126, miR-144, miR-223, miR-191, and miR-486-5p correlated inversely with insulin sensitivity index. In an Iraqi cohort, miR-197 (but not the miRNAs noted in the Swedish cohort) correlated positively with insulin sensitivity index, thus suggesting ethnicity-specific associations [86]. Overall, specific miRNA signatures have the capability to predict future development of T2D. Ethnicity-specific miRNA signatures should be established to improve the predictive potential of these signatures.

### 3.3. miRNA Biomarkers in Gestational Diabetes

**Figure 3:**
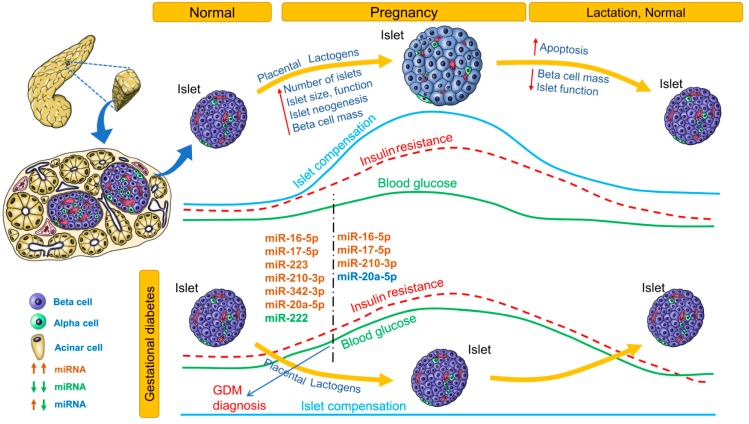
Circulating miRNA profile at different stages of gestational diabetes. Changes in blood glucose, insulin resistance, and islet compensation over the course of normal pregnancy/lactation and gestational diabetes mellitus (GDM) pathophysiology are provided as line profiles. Circulating miRNA profiles identified and consistent in at least 2 clinical studies are provided. Upregulated miRNAs are shown in red; downregulated miRNAs, in green; upregulated or downregulated in at least 2 studies, in blue.

Changes in blood glucose, insulin resistance, islet compensation and circulating miRNA profiles in GDM are summarized in Figure 3. Only miRNAs that were identified independently in at least 2 studies are listed. For an extensive list of all differentially expressed miRNAs, see Table 4. The association (positive or negative) of differentially expressed miRNAs with metabolic parameters is provided in Supplementary Table S1.

During pregnancy, insulin resistance imposes increased metabolic demand on islets. Islet adaptation to pregnancy is crucial not only for maintaining glucose homeostasis but also for preventing excessive nutrient flow from mother to fetus across the placenta. Placental lactogens induce beta-cell mass expansion, and islet neogenesis (in humans) has been demonstrated [96]. Lack of islet adaptation to pregnancy results in GDM, the incidence of which is steadily increasing. GDM also increases the risk of future development of T2D in both mother and child. Glucose tolerance is usually tested between 24 and 28 weeks of pregnancy. By this time, blood glucose levels are already significantly elevated and may affect the developing fetus. There is a need for predicting development of GDM earlier in pregnancy, so that intervention strategies can be applied before blood glucose levels increase.

Table 4 lists the miRNA profiles identified in various studies at different stages of gestation. Of these miRNA profiles, the most consistently upregulated miRNAs before 20 weeks of pregnancy were miR-16-5p, miR-17-5p, miR-223, miR-210-3p, miR-342-3p, and miR-20a-5p, whereas miR-222 was the most consistently downregulated miRNA (Figure 3). In women 16.1 weeks’ pregnant, higher levels of miR-155-5p and miR-21-3p were associated with higher odds for GDM. In women with male offspring (but not female offspring), miR-155-5p, miR-21-3p, miR-146b-5p, miR-223-3p, miR-517-5p, and miR-29a-3p were associated with GDM risk [101]. In pregnant women, miR-30a-5p, miR-130a, and miR-150 were associated with higher pregnancy weight gain, whereas miR-99b, miR-103, mIR-128a, miR-221, miR-324-3p, and miR-652 were associated with lower pregnancy weight gain. In pregnant women who were followed up to the 28th week, levels of miR-16-5p, miR-17-5p, and miR-20a-5p were positively correlated with insulin resistance (HOMA-IR) [100]. In pregnant women with GDM, high levels of miR-330-3p were inversely associated with insulinemia. In contrast, in GDM patients with lower miR-330-3p levels but levels higher than those of control women, insulinemia was higher than in the group with high miR-330-3p levels. Moreover, women with high miR-330-3p levels had an aggressive diabetic phenotype among GDM patients [102]. In pregnant South African women, miR-20a-5p and one or more risk factors were significant predictors of GDM [105]. The predictive potential for these miRNAs should be validated in further studies.

### 3.4. Common miRNA Signatures for Diabetes

**Figure 4:**
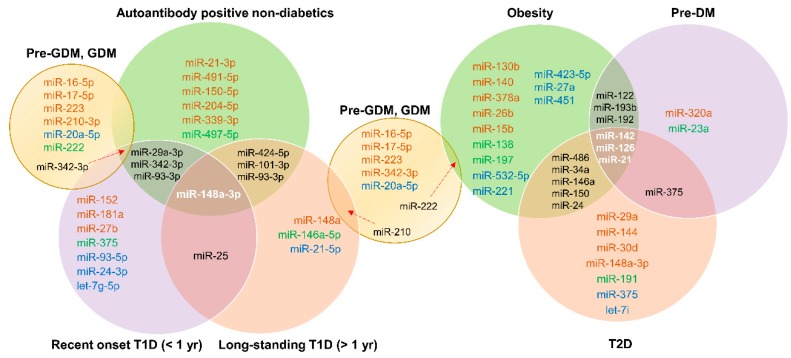
Common miRNA signatures. Venn diagrams indicate common miRNA signatures between different stages/types of diabetes. For expression of these miRNAs in specific disease states, refer to Figure 1, Figure 2 and Figure 3 and Table 1, Table 2, Table 3 and Table 4.

Figure 4 depicts common miRNA signatures for those at risk for T1D, with recent-onset T1D, longstanding T1D, obesity, pre-DM, T2D, and GDM. For specific upregulation or downregulation of these common miRNAs in specific stages, see Table 1, Table 2, Table 3 and Table 4. miR-148a-3p was common in all stages of T1D, suggesting its potential as an early biomarker specific to T1D pathogenesis. Among those at risk for T1D and with recent-onset T1D, miR-29a-3p, miR-342-3p, and miR-93-3p were common. miR-25 was common in individuals with recent-onset or longstanding T1D. miRNAs common to obesity, pre-DM, and T2D were miR-142, miR-126, and miR-21. miR-375 was common to both pre-DM and T2D patients, suggesting early involvement of this miRNA in the pathogenesis of T2D. miR-342-3p was common to those with GDM, those at risk for T1D, and patients with T1D, whereas miR-210 was shared by individuals with GDM and longstanding T1D. Thus, there appear to be some common signatures, but more studies are needed to make solid connections between various types of diabetes.

### 3.5. Tissue of Origin and Functional Significance

The tissue of origin of circulating miRNAs is important to establish islet stress-specific signatures. However, such determinations are difficult to implement as miRNAs have diverse roles in different tissues in a context-dependent manner and thus are expressed in multiple tissues with differing expression patterns. For example, miR-375 is abundantly expressed in islets but is predominantly expressed in the pituitary gland. Although islets are important for glucose and energy homeostasis, coordinated actions of other organs including the brain, liver, adipose tissue, and muscle are central to regulation of whole-body metabolism. From our list of consistently up- or down-regulated miRNAs, we observed that miR-150-5p, identified in the circulation in non-diabetic individuals with autoantibodies, obesity, or T2D, was the only miRNA with predominant expression in the pancreas [109]. Nevertheless, irrespective of tissue source, miRNA signatures specific to early stages of diabetes are important for distinguishing diabetes-prone individuals from the healthy population. The functional significance of circulating miRNAs is unclear, with many reports suggesting roles in communication between different tissues. However, circulating miRNAs may also be a result of a specific pathological event in the course of disease. For example, reduced miR-126 in circulation in pre-DM and T2D signifies endothelial dysfunction (caused by hyperglycemia exposure) and correlates with subclinical and manifest peripheral artery disease [39]. Further investigations are needed to delineate functional roles of circulating miRNA signatures in diabetes.

### 3.6. Challenges in Establishing miRNA Biomarkers

Despite careful analysis of a number of studies, our literature review showed only a handful of consistent miRNA signatures. It is possible to miss important miRNA signatures because of technical differences in sample handling, miRNA measurement, and data analysis. As for differences in serum and plasma, the contribution of platelets to miRNA content should be addressed. Circulating miRNAs should ideally be cell free and reflect a true pathological state. Some of the studies reported here used sample pools for analyses which is not ideal for any analyses. Sample pools do not provide information on variability, with assumptions that all individuals in a cohort are identical in their disease state. Sample size is an important factor in determining true miRNA signatures for a particular disease state. We included studies with both small and large sample sizes in our analyses because it is important to establish consistency in miRNA signatures across independent centers. For example, miR-148a was elevated in a cohort of 16 newly diagnosed T1D patients (Seyhan et al., 2016) but this observation was also seen in large cohorts of 275 recent onset T1D patients (Nielsen et al., 2012). Thus including all studies in our analyses give us the opportunity to optimize and tailor future investigations to gaps in literature. In addition, in the studies we analyzed, normalization strategies varied from using endogenous miRNA controls to exogenous spike-in controls. While endogenous housekeeping controls are standard for gene expression analysis using qPCR, one needs to be careful before applying the same controls in measuring circulating miRNAs. For example, one of the studies used miR-191 as an endogenous control for normalization [43], but later studies confirmed that pre-DM and obesity may alter the circulating levels of miR-191 [37,64,78]. On the other hand, exogenous spike-in controls are used for handling technical differences in miRNA extraction and cDNA conversion. In addition, currently existing commercial kits recommend normalizing data to sample input (plasma or serum volume) rather than RNA concentration for cDNA input (as is the norm for qPCR). This is mainly because of difficulties in determining miRNA concentrations. Even though small RNA concentrations can be determined, miRNA fraction of the small RNAs may vary between samples. Such differences in small RNA input for cDNA conversion may introduce bias in the analysis. In our experience, we observed that in normal healthy conditions, the miRNA fraction of circulating small RNAs is lower and hence normalizing input for cDNA to sample volume may introduce bias in data analysis. Small RNA sequencing avoids this bias by normalizing the expression to number of reads instead of the sample input. However, this approach is not practical, as small RNA sequencing is too expensive to perform on a routine basis. Absolute quantification of miRNA concentrations using synthetic miRNA mimics can help overcome these problems. Absolute quantification will also help in comparing different data sets and in correlation analysis. To conclude, miRNA expression methodology should be standardized for possible clinical applications.

### 3.7. Future Directions and Conclusions

We have provided comprehensive information on the potential use of miRNA signatures selective for T1D, pre-DM, T2D, and GDM. Although islet-specific miRNAs are of special interest for identifying islet stress or damage, miRNA signatures not specific to islets may also be important in understanding the metabolic disarray involving multiple tissues in diabetes. Consistent miRNA signatures specific to different stages, identified especially in those studies involving non-diabetic individuals who developed pre-DM or T1D at follow-up, should be validated independently. In future studies, influences on circulating miRNA signatures by gender, age and other factors (other diseases, medications, lifestyle factors) should be investigated. These studies will help establish miRNA signatures that can be used clinically to predict diabetes in the general population. Further, amassing data samples, standardizing miRNA detection technologies, and tracking and validating correlations with disease states will improve their predictive and diagnostic efficacy for developing strategies of therapeutic intervention.

## Figures and Tables

**Table 1:** miRNAs differentially expressed in patients with or at risk of type 1 diabetes (T1D).

miRNAs	+/−	Sample	Patient Classifications	Ethnicity	Method	Cohort Size *	Refs
Type 1 diabetes (recent onset)							
miR-152, miR-30a-5p, miR-181a, miR-24, miR-148a, miR-210, miR-27a, miR-29a, miR-26a, miR-27b, miR-25, miR-200a	+++	Serum	Recent-onset T1D	Hvidoere cohort	Small RNA seq, qPCR	P-275 C-151	[20]
miR-30a-5p, miR-181a, miR-26a, miR-25	+++	Serum	Recent-onset T1D	Danish remission cohort	Small RNA seq, qPCR	P-129 C-151	[20]
miR-375	−−−	Serum	Newly diagnosed T1D	Not available	qPCR	P-22 C-10	[24]
miR-454-3p, miR-222-3p, miR-144-5p, miR-345-5p, miR-125a-3p, miR-24-3p, miR-502-3p, miR-25-3p, miR-500a-5p, miR-324-5p, miR-140-5p, miR-192-5p, miR-331-3p, miR-377-3p, miR-221-3p, miR-182-5p, miR-103a-2-5p, miR-183-5p, let-7e-5p, miR-30e-5p, let-7g-5p, miR-18a-5p, miR-324-3p, miR-1468, miR-214-5p, miR-23b-3p, miR-93-5p	+++	Serum	Recent-onset T1D (<42 d)	Not available	qPCR	P-29 C-32	[25]
miR-375	NC	Serum	Recent-onset T1D (<42 d)	Not available	qPCR	P-29 C-32	[25]
miR-720, miR-636, miR-630, miR-490-5p, miR-154-3p, miR-675-3p, miR-100-5p, miR-639	−−−	Serum	Recent-onset T1D (<42 d)	Not available	qPCR	P-29 C-32	[25]
hsa-miR-24-3p, hsa-miR-146a-5p, hsa-miR-194-5p, hsa-miR-197-3p, hsa-miR-301a-3p, hsa-miR-375	NA	Plasma	Newly diagnosed T1D; measured at diagnosis predicts C-peptide 6-12 mo after diagnosis	Danish remission phase cohort	qPCR	P-123 No controls; follow-up study	[21]
miR-197-3p	NA	Plasma	Newly diagnosed T1D; measured 3 months after diagnosis predicted C-peptide at 12 mo	Danish remission phase cohort	qPCR	P-123 No controls; follow-up study	[21]
miR-122-5p, miR-125b-5p, miR-136-5p, miR-34a-5p, miR-342-3p, miR-152, miR-320b, miR-28-5p, miR-151a-3p, miR-181a-5p, miR-151a-5p, miR-423-5p, miR-199a-3p, miR-126-3p, miR-652-3p, miR-148b-3p, miR-27b-3p	+++	Serum	Recent-onset T1D	Not available	qPCR	P-8 C-17	[17]
miR-107, miR-22-3p, miR-590-5p, let-7g-5p, miR-24-3p, miR-32-5p, miR-22-5p, miR-16-2-3p, miR-93-5p, miR-25-3p, miR-140-3p, miR-19a-3p, miR-19b-3p, miR-16-5p, miR-30e-5p, miR-363-3p, miR-222-3p, miR-144-3p, miR-140-5p, miR-144-5p	−−−	Serum	Recent-onset T1D	Not available	qPCR	P-8 C-17	[17]
let-7g-5p, miR-24-3p	−−−	Serum	T1D	Not available	qPCR	P-10 C-10	[27]
miR-1225-5p, miR-320c	+++	Serum	Recent-onset T1D; duration <1 y	Not available	Microarray, qPCR	P-73 C-85	[28]
Type 1 diabetes (>1 y), LADA, MODY							
miR-21, miR-210	+++	Plasma, urine	Pediatric T1D; duration >1 y	Not available	qPCR	P-68 C-79	[29]
miR-126	+++	Urine	Pediatric T1D; duration >1 y	Not available	qPCR	P-68 C-79	[29]
miR-224	+++	Urine	*HNF1A* carriers (MODY), T1D	Not available	qPCR	P-38, 44 C-26	[30]
miR-148a, miR-21, miR-375	+++	Plasma	T1D, diagnosed before age 30 y	Not available	qPCR	P-16 C-27	[26]
miR-16, miR-302d-3p, miR-378e, miR-570–3p, miR-574-5p, miR-579	−−−	Plasma exosomes	T1D; duration of disease-25 y	Not available	Microarray	P-36 C-36	[22]
miR-25-3p	+++	Plasma exosomes	T1D; duration of disease-25 y	Not available	Microarray	P-36 C-36	[22]
miR-21-5p, miR-101-3p, miR-103a-3p, miR-148b-3p, miR-155-5p, miR-200a-3p, miR-210-3p, miR-1275	+++	Plasma	Recent-onset T1D; duration of first group < 5 y and second group > 5 y; levels in second group normalized to control levels	Not available	qPCR	Group 1 P-29 Group 2 P-31, C-40	[31]
miR-146a-5p	−−−	Plasma	Recent-onset T1D; duration of first group < 5 y and second group > 5 y; levels in second group normalized to control levels	Not available	qPCR	Group 1 P-29 Group 2 P-31, C-40	[31]
miR-21-5p, miR-148a	+++	Serum	T1D, duration of disease 15.7 ± 11.3 y	Not available	qPCR	P-15 C-14	[32]
let-7g-5p, miR-24-3p	−−−	Serum	C-peptide negative *GCK-MODY*	Not available	qPCR	P-13 C-10	[27]
miR-424, miR-218	+++	Urine exosomes	T1D	Not available	No access; Abstract only	No access; abstract only	[33]
miR-21, miR-25, miR-146a, miR-181a	−−−	Serum	LADA and T1D; > 1 y after diagnosis	Not available	qPCR	T1D P-29 LADA P-16 C-19	[23]
miR-555, miR-93-5p	+++	Plasma	LADA	Not available	Microarray, qPCR	P-12 C-6	[34]
miR-507, miR-517a-3p, miR-517b-3p, miR-4691-3p, miR-448, miR-370-5p, miR-1236-3p, miR-1267	−−−	Plasma	LADA	Not available	Microarray, qPCR	P-12 C-6	[34]
Non-diabetic autoantibody-positive children compared with T1D children							
miR-21-3p, miR-424-5p, miR-29a-3p, miR-150-5p, miR-342-3p, miR-491-5p	+++	Serum	In relatives of T1D individuals who were autoantibody positive and progressed to develop T1D in 1.8 ± 1.9 y at follow up; controls were nonprogressors but autoantibody positive	T1D TrialNet Pathway to Prevention study cohort	qPCR	300 children	[16]
miR-339-3p, miR-148a-3p	+++	Serum	Autoantibody-positive children, high risk for T1D. miR-148a-3p levels in comparison to low HLA-risk children among the high-risk group.	All Babies in Southeast Sweden cohort	qPCR	P-21 C-17	[17]
miR-497-5p, miR-93-3p	−−−	Serum	Autoantibody-positive children, high risk for T1D. miR-93-3p levels in comparison to low HLA-risk children among the high-risk group.	All Babies in Southeast Sweden cohort	qPCR	P-21 from 17,055 participants C-17	[17]
miR-101-3p	+++	Serum	In non-diabetic individuals with single or multiple autoantibody and T1D	Not available	qPCR	P-26, 12 C-43	[18]
miR-204-5p	+++	Serum	Elevated immediately after islet autotransplantation; pediatric recent-onset T1D; adult at-risk subjects with positive autoantibody and recent-onset T1D.	TrialNet Pathway to Prevention cohort and center recruits	qPCR	P-14 C-10	[19]

* P and C indicate patient and control sample size, respectively. LADA, latent autoimmune diabetes in adults; MODY, maturity-onset diabetes of the young; NC, no change; qPCR, quantitative polymerase chain reaction; seq, sequencing; T1D, type 1 diabetes.

**Table 2:** miRNAs differentially expressed in pre-DM, obese, and non-diabetic individuals at follow-up.

miRNAs	+/−	Sample	Patient Classifications	Ethnicity	Method	Cohort Size *	Ref
miR-15a, miR-29b, miR-126, miR-223	−−−	Plasma	Non-diabetic individuals who developed T2D in 10 y	Bruneck study, Italy	miRNA qPCR arrays	P –19 C-822	[39]
miR-28-3p	+++	Plasma	Non-diabetic individuals who developed T2D in 10 y	Bruneck study, Italy	miRNA qPCR arrays	P-19 C-822	[39]
miR-15b	+++	Serum	Obesity	Not available	qPCR	P-20 C-20	[43]
miR-138, miR-376a	−−−	Serum	Obesity	Not available	qPCR	P-20 C-20	[43]
miR-30c, miR-103, miR-191, miR-423-3p	NC	Serum	Obesity (used as internal controls)	Not available	qPCR	P-20 C-20	[43]
miR-16-1, miR-122, miR-130b, miR-140-5p, miR-142-3p, miR-222, miR-363, miR-423-5p, miR-486-3p, miR-486-5p, miR-532-5p	+++	Plasma	Childhood obesity	Caucasian	qPCR	P-40 C-85	[36]
miR-28-3p, miR-125b, miR-221, miR-328	−−−	Plasma	Childhood obesity	Caucasian	qPCR	P-40 C-85	[36]
miR-130b	+++	Serum	Obesity	Chinese	qPCR	P-44	[44]
let-7g, miR-221	+++	Serum	MetS	Chinese women	qPCR	P-31 C-71	[45]
miR-126	−−−	Plasma	T2D-susceptible individuals	Han Chinese	qPCR	P-30, 30 C-30	[46]
miR-126	−−−	Serum	IGT/IFG	Not available	qPCR	P-82 IGT, 75 IFG C-138	[47]
miR-23a	−−−	Serum	Pre-DM (IFG and IGT)	Han Chinese	Solexa seq, qPCR	P-20 C-20	[48]
miR-370, miR-378a-5p, miR-27a-5p	+++	Serum	Obese children and adolescents	Turkish	qPCR	P-45, 45 C-41, 41	[49]
miR-335-5p, miR-143-5p, miR-758-3p	−−−	Serum	Obese children and adolescents	Turkish	qPCR	P-45, 45 C-41, 41	[49]
miR-191-5p	+++	Plasma	Prevalent glycemic impairment (IGT, IFG, or T2D) but not on medications	Asian Indians	Firefly circulating miRNA assay	P-149	[37]
miR-122, miR-15a, miR-197, miR-320a, miR-423, miR-486	−−−	Plasma	Progressive glycemic impairment at 2.5-y follow-up (IGT, IFG, or T2D) but not on medications	Asian Indians	Firefly circulating miRNA assay	P-149	[37]
miR-138, miR-192, miR-193b, miR-214, miR-370, miR-375	ND	Plasma	Prevalent or progressive glycemic impairment at 2.5-y follow-up (IGT, IFG, or T2D) but not on medications	Asian Indians	Firefly circulating miRNA assay	P-149	[37]
miR-193b-3p, miR-22-3p, miR-320a, miR-486-5p	+++	Plasma	Non-diabetic healthy individuals with or without insulin resistance; levels higher in insulin-resistant group	50% Caucasian, 13% Asian, 8% African American, 5% Latino	Firefly circulating miRNA assay	93	[38]
miR-375	−−−	Plasma	Non-diabetic healthy individuals with or without insulin resistance; levels lower in insulin-resistant group	50% Caucasian, 13% Asian, 8% African American, 5% Latino	Firefly circulating miRNA assay	93	[38]
miR-20b-5p, miR-214-3p, miR-22-3p, miR-486-5p	+++	Plasma	TZD intervention study: Non-diabetic healthy individuals with insulin resistance; TZD intervention responder levels	50% Caucasian, 13% Asian, 8% African American, 5% Latino	Firefly circulating miRNA assay	93	[38]
miR-21-5p, miR-320a	−−−	Plasma	TZD intervention study: Non-diabetic healthy individuals with insulin resistance; TZD intervention responder levels	50% Caucasian, 13% Asian, 8% African American, 5% Latino	Firefly circulating miRNA assay	93	[38]
miR-128, miR-99b-5p	+++	Serum	IGT patients	Asian Indian	miRNA human panel I qPCR	P-47 C-49	[50]
miR-423-5p	−−−	Serum	IGT patients	Asian Indian	miRNA human panel I qPCR	P-47 C-49	[50]
miR-192, miR-193b	+++	Serum	Pre-diabetic (Pre-DM) patients (IFG, IGT); normalized by exercise intervention	Not available	qPCR	P-22, 21 C-29	[51]
miR-152, miR-17, miR-593	+++	Plasma	Obesity	Not available	qPCR	25/group	[52]
miR-138	−−−	Plasma	Obesit	Not available	qPCR	25/group	[52]
miR-126	−−−	Plasma	Normoglycemic individuals who developed T2D at 2-y follow-up	Han Chinese	qPCR	20/group	[40]
miR-31-5p, miR-2355-5p, miR-26b-5p	−−−	Plasma	Overweight/obese children and adolescents	I.Family study; Italian cohort	qPCR arrays	P-10 C-10	[53]
miR-320a, miR-1231, miR-361-3p, miR-136-5p, miR-206	+++	Plasma	Overweight/obese children and adolescents	I.Family study; Italian cohort	qPCR arrays	P-10 C-10	[53]
miR-29b, miR-126, miR-155	−−−	Serum	Pre-DM	ORIGINS trial	qPCR	P-21 C-20	[54]
miR-21, miR-24, miR-27a, miR-34a, miR-126, miR-146a	+++	Serum	Obesity	ORIGINS trial	qPCR	P-21 C-20	[54]
miR-25, miR-93	−−−	Serum	Obesity	ORIGINS trial	qPCR	P-21 C-20	[54]
miR-935	+++	Plasma	Non-diabetic obese adults; 16-wk weight loss intervention (diet and exercise) study; low responder group high levels	Not available	qPCR	111	[55]
miR-126	−−−	Serum	IGT	Egyptian	qPCR	P-86 C-100	[56]
miR-126, miR-146a	−−−	Plasma	Pre-DM patients	Not available	qPCR	P-12 C-27	[26]
miR-1249, miR-320b	−−−	Plasma	IGT/IFG	Han Chinese	Solexa seq, qPCR	3/group-seq 50/group-qPCR	[57]
miR-6069, miR-572	+++	Plasma	IGT/IFG and T2D; miR-6069 only in IFG/IGT	Han Chinese	Solexa seq, qPCR	3/group-seq 50/group-qPCR	[57]
miR-181a, miR-323-3p, miR-342-3p, miR-222, miR-483-5p, miR-151-5p, miR-532-3p, miR-142-5p, miR-625, miR-27b, miR-590-3p	−−−	Plasma	Non-diabetic, normotensive; prognostic and diagnostic biomarkers of beta-cell dysfunction in individuals who proceeded to develop pre-DM at 3-y follow-up	European RISC cohort	qPCR-based miRNA card	Total-1384 P-90 IGT at 3-y follow-up	[41]
miR-21, miR-145, miR-151-3p, miR-134, miR-215, miR-590-3p, miR-485-3p, miR-181a, miR-323-3p	−−−	Plasma	Non-diabetic, normotensive; diagnostic biomarkers of beta-cell dysfunction in individuals who developed pre-DM at 3-y follow-up	European RISC cohort	qPCR-based miRNA card	Total-1384 P-90 IGT at 3-y follow-up	[41]
miR-122-5p, miR-99a-5p	+++	Plasma	IGT	DIAPASON study cohort	qPCR miRNA panels	P-9 C-9	[58]
miR-18a-5p, miR-18b-5p, miR-30d-5p, miR-23a-3p, miR-24-3p, miR-27a-3p, miR-28-3p	−−−	Plasma	IGT	DIAPASON study cohort	qPCR miRNA panels	P-9 C-9	[58]
miR-326, miR-24, miR-425, miR-652	+++	Plasma	After weight loss intervention in MetS patients	PRYSMS study	Firefly circulating miRNA assay	171	[59]
miR-106b, miR-140, miR-20b, miR-363, miR486, miR-532, miR-92a, miR-93, miR-let7c	−−−	Plasma	After weight loss intervention in MetS patients	PRYSMS study	Firefly circulating miRNA assay	171	[59]
miR-126-3p	−−−	Microparticles	Pre-DM	Not available	qPCR	P-39 C-53	[60]
miR-320a, miR-197-3p, miR-23-3p, miR-221-3p, miR-27a-3p, miR-130a-3p	−−−	Serum	Obese individuals with or without MetS; these levels were further reduced to negligible in MetS patients	Not available	qPCR	Obese-17 MetS-16 C-24	[61]
miR-144, miR-365, miR-32, miR-451, miR-150	+++	Plasma	Obese, insulin sensitive	European	qPCR miRNA panels	P-11, 19 C-12	[62]
Let-7f, let-7e, miR-409-3p, miR-151-5p, miR-374b	−−−	Plasma	Obese, insulin sensitive	European	qPCR miRNA panels	P-11, 19 C-12	[62]
miR-144, miR-193b, miR-365, miR-451, miR-122	+++	Plasma	Obese, insulin resistant	European	qPCR miRNA panels	P-11, 19 C-12	[62]
miR-409-3p, let-7f, let-7e, miR-1974, miR-382	−−−	Plasma	Obese, insulin resistant	European	qPCR miRNA panels	P-11, 19 C-12	[62]
miR-7, miR-192	+++	Plasma	Pre-DM; vitamin D supplementation reduced levels after 6 mo	CaDDM study	miRNA qPCR arrays	21/group	[63]
miR-152	−−−	Plasma	Pre-DM; vitamin D supplementation increased levels after 6 mo	CaDDM study	miRNA qPCR arrays	21/group	[63]
miR-199a-5p, miR-122-5p, miR-191-5p, miR-27b-3p, miR-15b-5p, miR-222-3p, miR-223-3p, miR-181b-5p, miR-23a-3p, miR-21-5p, miR-34a-5p, miR-192-5p, miR-29a-3p, miR-214-5p, miR-155-5p, miR-103a-5p	+++	Plasma	Obese children with NAFLD	Not available	qPCR	P-20 C-10	[64]
MiR-451, miR-16, miR-150, miR-375	NC	Plasma	Obese children with NAFLD	Not available	qPCR	P-20 C-10	[64]
miR-122	+++	Serum, plasma	General public, followed up for 15 y, MetS	Bruneck study	qPCR	1000	[42]
miR-125b, miR-378a, miR-192, miR-629, miR-22-5p, miR-15a	−−−	Plasma	miRNA levels after bariatric surgery	Not available	miRNA qPCR panels	9	[65]
miR-126, miR-130b, miR-221, miR-222	+++	Plasma	Obese, non-diabetic	Not available	qPCR	P-12 C-12	[66]
miR-21, miR-126, miR-130b, miR-221, miR-222	+++	Plasma	Exercise intervention further increased these levels	Not available	qPCR	P-12 C-12	[66]
miR-222, miR-486, miR-146b, miR-15b, miR-146a, miR-20a, miR-26b	+++	Serum	Obesity	Not available	Small RNA seq, qPCR	P-206, 101 C-146, 82	[67]
miR-197	−−−	Serum	Obesity	Not available	Small RNA seq, qPCR	P-206, 101 C-146, 82	[67]
miR-21	−−−	Serum	Obese non-diabetic	Not available	qPCR	P-45 C-42	[68]
miR-150	+++	Plasma	Pre-DM or progressed to pre-DM, 5-y follow-up study; baseline levels	CORDIOPREV study	qPCR	462	[69]
let-7b, miR-144, miR-29a	+++	Plasma	IFG	Han Chinese	Microarray, qPCR	P-72 C-94	[70]
miR-142	−−−	Plasma	IFG	Han Chinese	Microarray, qPCR	P-72 C-94	[70]
miR-758-3p, miR-29b-3p	+++	Plasma	Detected in obesity but undetected in MetS	DairyHealth study, Denmark	MicroRNA qPCR panel, qPCR	26/group	[71]
miR-146a-5p, miR-126	+++	Plasma	Obese non-diabetic; exercise intervention reduced levels of miR-146a	CURIAMO trial cohort	qPCR	P-31 C-31	[72]
miR-142-3p, miR-140-5p, miR-222, miR-143, miR-130	+++	Plasma	Overweight and obese non-diabetic children (12-18 y)	Not available	qPCR	P-100, 100 C-50	[73]
miR-532-5p, miR-423-5p, miR-520c-3p, miR-146a, miR-15a	−−−	Plasma	Overweight and obese non-diabetic children (12-18 y)	Not available	qPCR	P-100, 100 C-50	[73]
miR-122-5p, miR-193b-5p, miR-26b-3p, miR-4449, let-7a-3p, miR-1290, let-7f-1-3p, miR-193a-5p, miR-183-5p, miR-126-5p	+++	Serum exosomes	Obesity	Not available	Small RNA seq	P-16 C-18	[74]
miR-4461, miR-1273a, miR-6739-5p, miR-1273g-3p, miR-4284, miR-6751-3p, miR-4485-5p, miR-8485, miR-1285-3p, miR-20a-5p	−−−	Serum exosomes	Obesity	Not available	Small RNA seq	P-16 C-18	[74]
miR-1246, miR-1290, miR-193b-5p, miR-378c, miR-378d, miR-378g, miR-424-5p, miR-4449, miR-6126	−−−	Serum exosomes	Reduction in levels after bariatric surgery in obese patients	Not available	Small RNA seq	P-16 C-18	[74]
miR-92a		Serum	Obesity	Not available	qPCR	P-26 C-7	[75]
65 miRNAs	+++	Plasma	Obese insulin-resistant individuals	Chinese	MiRXES	P-9 C-9	[76]
73 miRNAs	−−−	Plasma	Obese insulin-resistant individuals	Chinese	MiRXES	P-9 C-9	[76]
miR-122-5p, miR-210-3p, miR-3200-3p, miR-376b-3p, miR-378a-3p, miR-4532-5p, miR-660-3p, miR-375, miR-192-5p, miR-127-5p	+++	Plasma	Pre-DM who progressed to T2D at 5-y follow-up	METSIM study	Small RNA seq, qPCR	P-290 (145 each)	[77]
miR-10b-5p, miR-191-3p, miR-215-5p, miR-501-5p, miR-551a, miR-874-3p	+++/−−−	Plasma	Overweight/obese children and adolescents	I.Family study; 8 European countries	qPCR arrays	P-95 C-95	[78]
miR-21	+++	Plasma	IGT	DIAPASON study cohort	qPCR	P-43 C-39	[79]
miR-103a	+++	Plasma	Pre-DM	Han Chinese	qPCR	P-47 C-50	[80]
miR-103b	−−−	Plasma	Pre-DM	Han Chinese	qPCR	P-47 C-50	[80]

* P and C indicate patient and control sample size, respectively. DM, diabetes mellitus; IFG, impaired fasting glucose; IGT, impaired glucose tolerance; MetS, metabolic syndrome; NAFLD, non-alcoholic fatty liver disease; NC, no change qPCR, quantitative polymerase chain reaction; seq, sequencing; TZD, thiazolidinedione. ORIGINS, birth cohort; DIAPASON, diabetes prediction and screening observational; PRYSMS, Practicing Restorative Yoga vs. Stretching for the Metabolic Syndrome; CaDDM, Calcium and Vitamin D for Diabetes Mellitus; CARDIOPREV, cardiovascular disease prevention; CURIAMO, Centro Universitario Ricerca Interdipartimentale Attività Motoria; METSIM, Metabolic Syndrome In Men.

**Table 3:** miRNAs differentially expressed in patients with type 2 diabetes.

miRNAs	+/−	Sample	Patient Classifications	Ethnicity	Method	Cohort Size *	Ref
miR-20b, miR-21, miR-24, miR-15a, miR-126, miR-191, miR-197, miR-223, miR-320, miR-486	−−−	Plasma	T2D	Bruneck study, Italy	miRNA qPCR arrays	822	[39]
miR-28-3p	+++	Plasma	T2D	Bruneck study, Italy	miRNA qPCR arrays	822	[39]
miR-503	+++	Plasma	T2D	Not available	qPCR	11/group	[81]
miR-9, miR-29a, miR-30d, miR-34a, miR-124a, miR-146a, miR-375	+++	Serum	T2D	Han Chinese	qPCR	P-9 C-12	[82]
miR-503	−−−	Serum	T2D	Not available	miRNA qPCR panels	P-13 C-20	[43]
miR-375	+++	Plasma	T2D	Chinese Kazak	qPCR	P-100 C-100	[83]
miR-126	−−−	Plasma	T2D-susceptible individuals, T2D	Han Chinese	qPCR	30/group	[46]
miR-146a	−−−	Serum	T2D	Not available	qPCR	P-56 C-40	[84]
miR-126	−−−	Serum	New T2D	Not available	qPCR	P-160 C-138	[47]
miR-199a	+++	Plasma	T2D	Chinese	qPCR	64/group	[85]
miR-23a, let-7i, miR-486, miR-96, miR-186, miR-191, miR-192, miR-146a	−−−	Serum	T2D	Han Chinese	Solexa seq, qPCR	P-20 C-20	[48]
miR-144, miR-486-5p	+++	Plasma	T2D	Swedes	qPCR	P-14 C-54	[86]
miR-150	+++	Plasma	T2D	Iraqi	qPCR	P-19 C-65	[86]
miR-375	+++	Plasma	Increased in T2D	Not available	qPCR	P-54 C-53	[87]
miR-103	+++	Urine	T2D	Not available	qPCR	P-36 C-26	[30]
miR-101, miR-375, miR-802	+++	Serum	T2D	Japanese	qPCR	P-155 C-49	[88]
Let-7d-3p, miR-128, miR-130b-3p	+++	Serum	T2D	Asian Indian	qPCR	P-49 C-49	[50]
miR-142-3p	−−−	Serum	T2D	Asian Indian	qPCR	P-49 C-49	[50]
miR-593	−−−	Plasma	T2D	Not available	qPCR	25/group	[52]
miR-21, miR-24, miR-34a, miR-148a, *miR-27a, miR-146a, miR-223, miR-326*	+++	Serum	T2D; miRNAs in italics were elevated in comparison to pre-T2D	ORIGINS trial	qPCR	P-17 C-20	[54]
miR-126	−−−	Serum	T2D	Egyptian	qPCR	P-100 C-100	[56]
miR-148a, miR-21, miR-30d, miR-34a	+++	Plasma	T2D	Not available	qPCR	P-31 C-27	[26]
miR-1249, miR-320b	−−−	Plasma	T2D	Han Chinese	Solexa seq, qPCR	3/group-seq 50/group-qPCR	[57]
miR-572	+++	Plasma	T2D	Han Chinese	Solexa seq, qPCR	3/group-seq 50/group-qPCR	[57]
miR-148a-3p	+++	Plasma	T2D	DIAPASON study cohort	qPCR miRNA panels	P-9 C-9	[58]
miR-222-3p, miR-342-3p	−−−	Plasma	T2D	DIAPASON study cohort	qPCR miRNA panels	P-9 C-9	[58]
miR-126-3p	−−−	Microparticles	T2D	Not available	qPCR	P-68 C-53	[60]
miR-144, miR-193b, miR-136, miR-34a, miR-32	+++	Plasma	Obese T2D	European	qPCR miRNA panels	P-11, 15 C-12	[62]
Let-7d, let-7c, let-7e, let-7f, miR-485-3p	−−−	Plasma	Obese T2D	European	qPCR miRNA panels	P-11, 15 C-12	[62]
miR-409-3p, miR-665, miR-766-3p	−−−	Serum	T2D	Chinese	miRNA qPCR array	P-10 C-5	[89]
miR-455-5p, miR-454-3p, miR-144-3p, miR-96-5p	+++	Serum	T2D	Chinese	miRNA qPCR array	P-10 C-5	[89]
miR-7	+++	Serum	T2D	Not available	qPCR	P-152 C-74	[90]
miR-122	+++	Serum and plasma	T2D	Bruneck study	qPCR	1000	[42]
miR-486, miR-146b, miR-15b	+++	Serum	T2D	Not available	Small RNA seq, qPCR	P-206, 101 C-146, 82	[67]
miR-21	−−−	Serum	Obese, T2D	Not available	qPCR	P-45 C-42	[68]
miR-150, miR-30a-5p	+++	Plasma	T2D	CORDIOPREV study	qPCR	462	[69]
miR-375	−−−	Plasma	T2D	CORDIOPREV study	qPCR	462	[69]
miR-144-3p, miR-155-5p, miR-29a-5p, let-7b-5p, let-7i-5p	+++	Plasma	Newly diagnosed T2D	Han Chinese	Microarray, qPCR	P-112 C-94	[70]
miR-142	−−−	Plasma	Newly diagnosed T2D	Han Chinese	Microarray, qPCR	P-112 C-94	[70]
miR-30d	+++	Plasma	T2D	Indian	qPCR	P-30 C-30	[91]
miR-141	+++	Serum	Elderly T2D (60–65 y)	Not available	qPCR	P-50	[92]
miR-7-5p, let-7f-5p, miR-15b-5p, miR-320c, miR-205-5p, miR-335-5p	+++	Plasma	Obese diabetes and levels after bariatric surgery	Not available	qPCR	29	[93]
let-7i-5p	−−−	Plasma	Obese diabetes and levels after bariatric surgery	Not available	qPCR	29	[93]
miR-21	+++	Plasma	T2D	DIAPASON study cohort	qPCR	P-27 C-39	[79]
miR-103a	+++	Plasma	T2D	Han Chinese	qPCR	P-48 C-50	[80]
miR-103b	−−−	Plasma	T2D	Han Chinese	qPCR	P-48 C-50	[80]
miR-183-5p, miR-486-3p	+++	Plasma	Recent-onset T2D; miR-486-3p only in men not women	Arab/Jewish Israeli population	qPCR	88	[94]
miR-423	−−−	Plasma	Recent-onset T2D	Arab/Jewish Israeli population	qPCR	88	[94]

* P and C indicate patient and control sample size, respectively. T2D indicates type 2 diabetes; qPCR, quantitative polymerase chain reaction.

**Table 4:** miRNAs differentially expressed in patients at risk for or with gestational diabetes mellitus.

miRNAs	+/−	Sample	Patient Classifications	Ethnicity	Method	Cohort Size *	Ref
miR-132, miR-29a, miR-222	−−−	Serum	16–19 weeks of gestation, before onset of GDM	Not available	miRNA qPCR panel, qPCR	24, 36, 16/group	[97]
miR-29c, miR-99b, miR-103, miR-221, miR-340, miR-122, miR-324-3p, miR-375, miR-652	−−−	Plasma	Gestational obesity patients	Caucasian	qPCR array, qPCR	P-25 C-25	[98]
miR-30a-5p, miR-130a, miR-150, miR-625	+++	Plasma	Gestational obesity patients	Caucasian	qPCR array, qPCR	P-25 C-25	[98]
miR-122, miR-324-3p, miR-375, miR-652	−−−	Plasma	Gestational obesity and pre-gestational obesity patients	Caucasian	qPCR array, qPCR	P-20, 25 C-25	[98]
miR-16-5p, miR-17-5p, miR-19a-3p, miR-19b-3p, miR-20a-5p	+++	Plasma	Every 4 weeks of gestation, before onset of GDM	Not available	Small RNA seq, qPCR	P-10 C-10	[99]
miR-16-5p, miR-17-5p, miR-20a-5p	+++	Plasma	16–20 weeks, before onset of GDM, 20–24 and 24–28 weeks	Not available	qPCR	P-85 C-72	[100]
miR-155-5p, miR-21-3p, miR-146b-5p, miR-210-3p, miR-223-3p, miR-517-5p	+++	Plasma	At 16.1 weeks of gestation followed up to 28 weeks, GDM	Hispanic, non-Hispanic, Asian, White, other	qPCR	P-36 C-80	[101]
miR-330-3p, miR-483-5p	+++	Plasma	24–33 weeks of gestation, GDM	Not available	miRNA array, qPCR	P-21 C-10	[102]
miR-183-3p, miR-200b-3p, miR-17-5p (trend), miR-125b-5p, miR-191-5p (trend), miR-1290	+++	Serum	First trimester of pregnancy in GDM	Not available	qPCR	P-67 C-74	[103]
miR-183-5p, miR-200b-3p	+++	Serum	Second trimester of pregnancy in GDM	Not available	qPCR	P-67 C-74	[103]
miR-128-5p	−−−	Serum	Second trimester of pregnancy in GDM	Not available	qPCR	P-67 C-74	[103]
miR-183-5p, miR-200b-3p	−−−	Serum	Third trimester of pregnancy in GDM	Not available	qPCR	P-67 C-74	[103]
miR-125a-3p, miR-99b-5p, miR-197-3p, miR-22-3p, miR-27b-3p, miR-200a-3p, miR-141-3p	+++	Plasma exosomes	End of pregnancy, GDM	Caucasian, Australian, Asian, Middle Eastern	qPCR	P-12 C-12	[104]
miR-20a-5p, miR-222-3p	−−−	Serum	13–31 weeks, GDM	South African	miRNA qPCR arrays	81	[105]
Let-7e-5p, let-7g-5p, miR-100-5p, miR-101-3p, miR-146a-5p, miR-18a-5p, miR-195-5p, miR-222-3p, miR-23b-3p, miR-30b-5p, miR-30c-5p, miR-30d-5p, miR-342-3p, miR-423-5p, miR-92a-3p	+++	Plasma	23–31 weeks of gestation, GDM	Not available	miRNA qPCR arrays, qPCR	P-13 C-9	[106]
miR-122-5p; miR-132-3p; miR-1323; miR-136-5p; miR-182-3p; miR-210-3p; miR-29a-3p; miR-29b-3p; miR-342-3p, miR-520h	+++	Serum exosomes	6–15 weeks of gestation, before onset of GDM	White	qPCR	P-23 C-46	[107]
miR-223, miR-23a	+++	Plasma	First trimester in GDM women	Not available	qPCR	Not available	[108]

* P and C indicate patient and control sample size, respectively. GDM indicates gestational diabetes mellitus; qPCR, quantitative polymerase chain reaction; seq, sequencing.

## Supplementary Materials

The following are available online at https://www.mdpi.com/2073-4409/8/12/1533/s1. Table S1: miRNA associations with metabolic parameters in different diabetes types.
