# Supplementary material for: MicroRNA Signatures as Future Biomarkers for Diagnosis of Diabetes States
Source: Cells. 2019 Nov 28;8(12):1533. doi: 10.3390/cells8121533 (PMC6953078; doi:10.3390/cells8121533)
Supplement: Supplementary file 1 [file cells-08-01533-s001.pdf]

Table S1 miRNA associations with metabolic parameters in different diabetes types

Parameter	T1D/at-risk for T1D	Obesity	Pre-DM	T2D	GDM	References
Body weight related parameters						
Adiponectin		miR-335, miR-758, miR-142-3p, miR-140-5p, miR-222, miR-143, miR-130, miR-532-5p, miR-423-5p, miR-520c-3p, miR-146a, miR-15a				Can et al 2015<br>Al-Rawaf et al 2019
BMI		miR-223-3p, miR-21-5p, miR-29a-3p, miR-150-5p, miR-103a-5p, miR-142-3p, miR-140-5p, miR-222 miR-130, miR-130b, miR-486, miR-146b, miR-146a, miR-20a, miR-15b, miR-26b, miR-370, miR-335, miR-378, miR-197, miR-143		miR-21		Can et al 2015<br>Thompson et al 2017<br>Al-Rawaf et al 2019<br>Wang et al 2013<br>Cui et al 2018, Ghorbani et al 2018
Fat mass		miR-155, miR-29b				Nunez-lopez et al 2016
FMI		miR-222, miR-486, miR-146b, miR-146a, miR-20a, miR-15b, miR-26b, miR-197				Cui et al 2018
Lean mass		miR-148a				Nunez-lopez et al 2016
Leptin		miR-142-3p, miR-140-5p, miR-222, miR-130, miR-532-5p, miR-423-5p, miR-520c-3p, miR-146a, miR-15a, miR-370, miR-335, miR-378, miR-143				Can et al 2015<br>Al-Rawaf et al 2019
Leptin/Adiponectin ratio		miR-142-3p, miR-140-5p, miR-222 miR-143, miR-130, miR-532-5p, miR-423-5p, miR-520c-3p, miR-146a, miR-15a				Al-Rawaf et al 2019
Waist circumference		miR-146a-5p				Russo et al 2018
Waist to hip ratio		miR-142-3p, miR-140-5p, miR-222 miR-143, miR-130				Al-Rawaf et al 2019
Glycemic parameters						

C-peptide	miR-424-5p, miR-29a-3p, miR-150-5p, miR-342-3p, miR-375					Snowhite et al 2017
C-peptide, fasting	miR-25, miR-424-5p, miR-150-5p					Snowhite et al 2017<br>Neilson et al 2012
C-peptide AUC				miR-24		Seyhan et al 2016
Glucose, fasting			miR-126	miR-126, miR-486, miR-146b, miR-15b	miR-340	Zhang et al 2013, Rezk et al 2016, Giannella et al 2017, Cui et al 2018, Carreras-Bodosa et al 2015
Glucose, postprandial		miR-34a	miR-21	miR-126		Nunez-lopez et al 2016, La Sala et al 2019
Glucose AUC			miR-148a	miR-126, miR-375, miR-21		De candia et al 2017, Seyhan et al 2016
HbA1c (%)	miR-140-3p, miR-29a-3p, miR-148a-3p, let-7b-3p, miR-222-3p, miR-23a-3p, miR-30c-5p, let-7f-5p, miR-151a-5p, miR-26b-5p, miR-139-5p, miR-25, miR-200a-3p, miR-155-5p	miR-22, miR-210	miR-130b-3p, miR-374a-5p, miR-30d	miR-130b-3p, miR-374a-5p, miR-374a-5p, miR-222-3p, miR-126,	miR-652	Akerman et al 2018<br>Neilson et al 2012<br>Assmann et al 2018<br>Prabu et al 2015, De candia et al 2017, Jones et al 2017, Carreras-Bodosa et al 2015
HOMA-B			miR-34a	miR-34a		De candia et al 2017, Seyhan et al 2016
HOMA-IR		miR-335, miR-143, miR-378, miR-34a	miR-374a-5p, miR-29a, miR-144, miR-20a, let-7b, miR-21, miR-142	miR-374a-5p	miR-16-5p, miR-17-5, miR-20a-5p	Can et al 2015<br>Prabu et al 2015, De candia et al 2017, Liang et al 2018, La Sala et al 2019, Cao et al 2017
IFG/IGT					miR-221	Carreras-Bodosa et al 2015
Insulin, fasting		miR-34a	miR-374a-5p	miR-374a-5p		Nunez-lopez et al 2016<br>Prabu et al 2015
Insulin		let-7d				Jones et al 2017
Insulin sensitivity index				miR-197, miR-15a, miR-29b, miR-24, miR-126, miR-144, miR-223, miR-191, miR-486-5p		Wang et al 2014,
Insulinogenic index				miR-146a		Seyhan et al 2016
Insulin resistance					miR-122, miR-375	Carreras-Bodosa et al 2015
Insulinemia					miR-330-3p	Sebastiani et al 2017a
OGTT				miR-148a-3p, miR-222-3p		De candia et al 2017

			Auto-antibodies			
GADA	miR-378a-3p, miR-101-3p					Akerman et al 2018 Santos et al 2019
IA2A	miR-144-5p, miR-342-3p, miR-378a-3p					Akerman et al 2018
IDAA1c	miR-200a-3p, miR-155-5p					Assmann et al 2018
ZnT8A (Trp)	miR-378a-3p					Akerman et al 2018
			Liver function parameters			
ALT		miR-122-5p, miR-192-5p				Thompson et al 2017
AST		miR-34a-5p, miR-122-5p				Thompson et al 2017
Fatty liver index			miR-192, miR-193b			Parrizas et al 2015
			Lipid profile parameters			
Cholesterol		miR-146a-5p	miR-128			Prabu et al 2015, Russo et al 2018
HDL-cholesterol		miR-335, miR-758, miR-34a	miR-423-5p		miR-122, miR-324-3p, miR-375	Can et al 2015 Prabu et al 2015, Carreras-Bodosa et al 2015
Triglycerides		miR-370, miR-335, miR-143, miR-378, miR-22, miR-210	miR-192, miR-193b, miR-30d		miR-122, miR-324-3p, miR-375	Can et al 2015 Parrizas et al 2015, De candia et al 2017, Jones et al 2017, Carreras-Bodosa et al 2015
			Others			
Diastolic blood pressure		miR-27a				Nunez-lopez et al 2016
QUICKI score		miR-34a				Nunez-lopez et al 2016

miRNAs in brown font indicate those that associate with the corresponding parameters positively. miRNAs in green font indicate those that associate with the corresponding parameters negatively. miRNAs in blue font indicate those that may associate with the corresponding parameters negatively or positively.
